# New way of Providing Care: the Role of Telemental Health

**DOI:** 10.1192/j.eurpsy.2022.147

**Published:** 2022-09-01

**Authors:** U. Volpe

**Affiliations:** Università Politecnica delle Marche, Unit Of Clinical Psychiatry, Department Of Clinical Neurosciences/dimsc, Ancona, Italy

**Keywords:** telemental health, e-mental health, digital psychiatry, digital therapeutics

## Abstract

Telemental health care can be defined as the delivery of mental health care services at distance, by using information and communication technologies for the exchange of valid information for diagnosis, treatment and prevention of mental illnesses, as well as for research and education in the field of clinical psychiatry. While telemental health care practice was long established in many countries, its development proceeded with some variability worldwide. Over the past months, however, the recent COVID-19 pandemic has abruptly spread telemental health care practice worldwide, mostly to ensure the provision of care and assistance to psychiatric patients in spite of the governmental social contact restrictions. Although the process of rapid implementation has often happened at different rates and with different quality standards, across the various countries and sites, a global increase of the use of digital technologies has been reported. On the other hand, such recent events have also sparked a real paradigm shift in mental health care, significantly expanding the scope of e-mental health, given the recent availability of newer tools of digital psychiatry. In more detail, the use of mobile phones applications, of social media, of immersive reality and of chatbots is now driving psychiatry towards envisioning a more hybrid form of psychiatric practice, which holds the potential to finally overcome the traditional gap between the unmet needs of psychiatric patients and the relative lack of services and resources in mental health care. Here, the research evidence and the most compelling implementation issues in digital psychiatry will be reviewed.

**Disclosure:**

No significant relationships.

